# 

**Published:** 1895-08-17

**Authors:** 


THE HOSPITAL. ABSOLUTELY PURE, therefore BEST. AUGUST 17TH, 1895.

CADBURY'S

COCOA

"Of absolute purity and freedom from alkali."

—*Braithwaite.*

CADBURY'S

COCOA

"The Typical Cocoa of English Manufacture. Absolutely Pure."—*The Analyst.*

### THE HOSPITAL

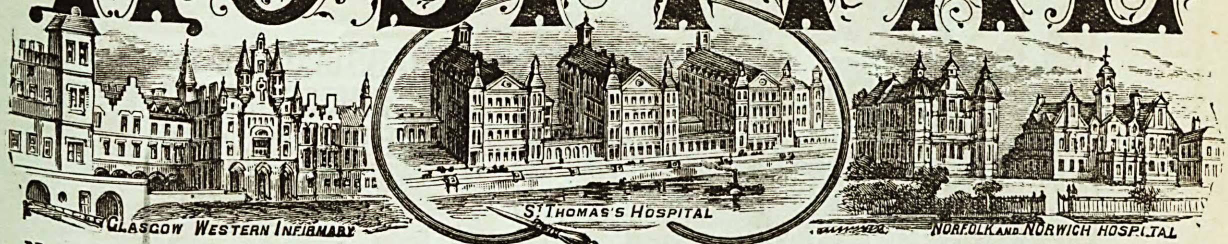

No. 464 Vol. XVIII.

SATURDAY, AUGUST 17TH, 1895.

WITH EXTRA NURSING SUPPLEMENT.
[Registered at the General Post Office as a Newspaper for transmission in the United Kingdom and Abroad.]

PRICE TWOPENCE.

Per Annum, 10s. 6d., post free.

Monthly Parts, 6d.; post free, 8d.

Ditto per Annum, 8s. 6d., post free.

